# Composition of Human Meibomian Gland Secretions: Insights from TOF-SIMS Analysis

**DOI:** 10.3390/ijms27031590

**Published:** 2026-02-05

**Authors:** Katarzyna Balin, Beata Węglarz, Karol Dobiczek, Dorota Tarnawska

**Affiliations:** 1 A. Chełkowski Institute of Physics, University of Silesia, 75 Pułku Piechoty 1A, 41-500 Chorzów, Poland; katarzyna.balin@us.edu.pl; 2 Clinical Department of Ophthalmology, District Railway Hospital, Panewnicka 65, 40-760 Katowice, Poland; 3 Institute of Biomedical Engineering, Faculty of Science and Technology, University of Silesia in Katowice, 75 Pułku Piechoty 1A, 41-500 Chorzów, Poland; 4 Department of Human-Centered Artificial Intelligence, Institute of Applied Computer Science, Jagiellonian University, Łojasiewicza 11, 30-348 Krakow, Poland; karol.dobiczek@gmail.com

**Keywords:** meibomian gland secretions, meibum, lipid analysis, mass spectroscopy, TOF-SIMS, PCA

## Abstract

This study evaluated the efficacy of the TOF-SIMS (time-of-flight secondary ion mass spectrometry) technique for the comprehensive lipidomic analysis of human meibum, a lipid-rich secretion essential for tear film stability, using samples collected from ten participants. The applied methodology proved effective in characterizing the complex chemistry of meibum, confirming the presence of diverse lipid classes, including fatty acids, sterols, and glycerolipids. Multivariate and pairwise statistical analyses, including permutational multivariate analysis of variance (PERMANOVA) and maximum mean discrepancy (MMD),confirmed the significant compositional difference between the two groups. Principal component analysis (PCA) revealed a clear separation between the samples, driven primarily by an elevated ratio of monounsaturated fatty acids (C18:1, C16:1) to cholesterol in the group with MGD compared to healthy controls. These findings demonstrate the utility of TOF-SIMS coupled with multivariate analysis for detecting disease-specific molecular alterations in meibum, highlighting its potential for differentiating ocular surface pathologies.

## 1. Introduction

### 1.1. Clinical Relevance of Meibum

Meibomian glands are specialized large, branched sebaceous glands embedded vertically within the tarsal plates of both upper and lower eyelids. Unlike the Zeiss glands, which are associated with eyelash follicles, Meibomian glands are independent of hair structures and open onto the eyelid margin, where they release their secretion onto the ocular surface [1,2]. Their secretion, also called meibum, forms the outer layer of the precorneal tear film, mainly preventing tear evaporation, but also protecting against tear leakage beyond the eyelid margin and ensuring eyelid glide during blinking [3,4]. Meibum, together with aqueous tears produced by lacrimal glands, forms the tear film [5]. The tear film consists of two main layers: an inner mucin–aqueous layer, composed of aqueous tears secreted by the main and accessory lacrimal glands, gel-forming mucins from conjunctival goblet cells, and membrane-associated mucins from corneal and conjunctival epithelial cells; and an outer lipid layer, primarily composed of meibum secreted by the Meibomian glands [2,3,4,5,6]. MGD causes qualitative or quantitative changes in glandular secretion and may result in alteration of the tear film, symptoms of eye irritation, clinically apparent inflammation, and ocular surface disease [7,8,9,10,11]. The biochemical profile of meibum is gaining attention as a source of clinically relevant biomarkers for ocular and systemic disorders. These include androgen metabolites reflecting intracrine activity [12], decreased levels of o-acyl-ω-hydroxy fatty acids (OAHFA) and cholesteryl esters in advanced MGD [13], elevated linoleic acid in severe MGD [14], altered cholesteryl esters/wax esters (CE/WE) ratios and simplified hydrocarbon chains in Sjögren’s syndrome [15], as well as reduced triglycerides (TG) and WE but increased CE in patients with type 2 diabetes and dry eye disease (DED) compared to non-diabetic DED [16,17]. Collectively, these molecular signatures highlight disease-specific lipid alterations that may assist in diagnosis and therapeutic evaluation. Beyond its role in secreting the lipid layer of the tear film, the meibomian gland is fundamental to ocular surface homeostasis, functioning both as a highly specialized sebaceous gland and as a source of molecular signaling molecules. Recent studies [18,19] have identified the MG as an endocrine-like organ for the ocular surface, primarily through its secretion of Ectodysplasin A (EDA). EDA plays a crucial role in maintaining corneal epithelial integrity by promoting proliferation and barrier function, as well as in supporting lacrimal gland development and function. A marked reduction in EDA in MGD patients provides a direct molecular link between MG dysfunction and ocular surface deterioration. Understanding the MG’s dual secretory role, producing both complex lipids and signaling proteins such as EDA, is therefore essential for elucidating the pathophysiology of MGD and its broader impact on ocular surface health. More recently, the detection of microplastics in meibum and their correlation with dry eye severity suggest that environmental exposures also leave measurable biochemical traces relevant to disease pathogenesis [20].

### 1.2. Current Understanding Meibum Composition

To gain a comprehensive understanding of meibum’s composition, structure, and function, a range of analytical techniques has been employed. The most commonly used techniques for analyzing meibum composition include mass spectrometry (MS), often coupled with high-performance liquid chromatography (HPLC-MS) or gas chromatography (GC-MS); nuclear magnetic resonance (NMR) spectroscopy for structural and quantitative lipid analysis; and vibrational methods such as infrared (IR) spectroscopy and Raman spectroscopy (RS) for conformational characterization. The literature data from various characterization methods indicate that meibum is a complex mixture comprising lipids (including phospholipids, triglycerides, wax esters, sterols, and fatty acids), proteins (such as albumin, immunoglobulins, lactoferrin, and lysozyme), carbohydrates (e.g., galactose and glucose), nucleotides, polyamines, water, and other minor components [14,16,17,21,22,23,24]. Differences in the reported composition often arise due to the inherent limitations of specific analytical techniques, which influence the detection and quantification of certain components. Analytical techniques such as mass spectrometry (MS) [25], frequently coupled with chromatographic methods (HPLC-MS or GC-MS) [15], represent the current standard in the lipidomic profiling of meibum. This approach enables the detection and characterization of intact complex lipid molecules, even at trace concentrations [25]. Gas chromatography (GC) is effective in analyzing wax esters (WE), which can often be examined without derivatization [26]. Nuclear magnetic resonance (NMR) spectroscopy provides quantitative information on lipid composition [27], including hydrocarbon chain branching, the degree of saturation, and molar ratios of specific lipid classes such as cholesterol esters (CE) to wax esters (WE). While infrared (IR) spectroscopy and Raman spectroscopy [25,26] enable conformational lipid analysis and class detection, they lack sufficient resolution for the definitive identification of individual lipid species in complex mixtures.

In addition to the limitations of specific experimental techniques, sample preparation procedures pose a significant challenge, as they can critically influence the accuracy and reliability of results. Maintaining sample integrity during meibum characterization requires careful handling, including the use of high-purity solvents to reduce contamination and inert atmospheres to prevent lipid oxidation or degradation. To prevent oxidation, samples can be stored in a dry state under, for example, argon atmosphere [28] and stored at low temperatures (e.g., −20 °C or −80 °C) [29]. A key aspect of meibum sample preparation is to avoid contact with plastic labware and silicone [17]. For example, chloroform, a commonly used solvent, is especially known for dissolving polymers, particularly silicone [26]. Many polymers and plasticizers can contaminate the sample even after brief contact [21]. It is recommended to use only glass, stainless steel, noble metals, or Teflon [17].

Sample preparation for MS involves collecting meibum using sterile, non-reactive tools to avoid contamination. The collected meibum is typically dissolved in an organic solvent mixture, usually chloroform–methanol (2:1 or 1:1, *v*/*v*), with vortexing or sonication to ensure complete dissolution. After centrifugation to remove insoluble debris, the solvent is evaporated under nitrogen or vacuum to obtain a dry lipid film. The dried extract is reconstituted in a compatible solvent, such as isopropanol (IPA) or methanol, often with gentle heating to aid dissolution. For GC-MS, derivatization (e.g., silylation) may be used to enhance volatility, and internal standards (e.g., deuterated lipids) are added for quantification. The sample is then introduced into the MS system via direct infusion (e.g., ESI or MALDI) or after separation by liquid chromatography (LC-MS) [17,30,31,32,33]. For gas chromatography (GC) and GC-MS, meibum is dissolved in solvents like hexane or chloroform, with derivatization often applied to increase the volatility of non-volatile lipids. In thin-layer chromatography (TLC), meibum is dissolved in chloroform–methanol (2:1, *v*/*v*), spotted onto a silica gel plate, and developed in a solvent system such as hexane–diethyl ether–acetic acid, followed by visualization using staining reagents [33,34]. Nuclear magnetic resonance (NMR) spectroscopy requires dissolving meibum in deuterated solvents like CDCl_3_, while Fourier-transform infrared (FTIR) spectroscopy involves spreading meibum onto an IR-transparent window (e.g., KBr or ZnSe) after dissolving it in a minimal amount of chloroform [22]. Raman spectroscopy, which provides molecular fingerprinting, typically analyzes meibum directly on a glass slide or substrate [35].

### 1.3. TOF-SIMS as a New Tool for Meibum Characterization

Despite their utility, commonly used techniques each have specific limitations, particularly related to sample preparation and to their ability to resolve structurally similar lipids and provide comprehensive compositional insights. Therefore, in the present study, we explored an analytical approach designed to overcome these limitations and enable a comprehensive characterization of the chemical composition of meibum. In this study, we focused on the surface-sensitive technique of time-of-flight secondary ion mass spectrometry (TOF-SIMS), which allows for precise determination of the composition and chemical structure of materials using minimal sample volumes. TOF-SIMS enables the direct and non-destructive analysis of very small amounts of native meibum, without the need for extraction, derivatization, or standards, which represents a major advantage compared to other analytical techniques. This is particularly important given that the typical dry weight of meibum collected from all four eyelids of a single donor is approximately 0.7 mg [36]. In a single measurement, this reveals signals from lipids, organic compounds, oxidation products, amino acid fragments, and inorganic ions. The technique simultaneously detects elemental and molecular ions at very low abundance (in the ppm range), covering a wide mass range from elemental species and small molecules to large lipids (up to 20,000 u), while also providing unique molecular mapping capabilities with high spatial resolution. While TOF-SIMS lacks the quantitative ability of techniques such as NMR or LC-MS, it uniquely enables the holistic molecular profiling of meibum composition and has the potential to identify markers of oxidative stress, inflammation, or ionic imbalance, which may be useful for discovering biomarkers of various diseases. TOF-SIMS is a well-established technique for lipid analysis in biological samples, with numerous studies demonstrating its effectiveness in characterizing the composition and spatial distribution of lipid species in cell membrane structures [37], various tissues [38,39], cancer, and other diseases [40,41]. TOF-SIMS has been successfully used to identify disease-specific biomarkers directly from biological samples. For instance, it has identified accumulated glycosphingolipids in Fabry disease [42], distinct phospholipid profiles in asthma from exhaled particles [43], and characteristic lipid alterations in melanoma tissue, enabling differentiation from healthy skin [44].

Given the importance of the precise molecular characterization of meibum in ocular surface health and disease, this study aimed to assess the utility of TOF-SIMS as a tool for rapid and comprehensive lipidomic analysis. The objectives were to establish a protocol for meibum profiling and to determine, using statistical approaches, whether the technique could identify distinct lipid signatures associated with MGD.

## 2. Results and Discussion

### 2.1. Compositional Analysis of Human Meibum

As a result of meibum measurements carried out using the TOF-SIMS technique, mass spectra with positive and negative polarities were analyzed. Analysis of these spectra revealed distinct chemical profiles, which is expected as the detection efficiency for ions is highly polarity-dependent. Selected mass range covers various molecular fragments, providing insight into the distribution and abundance of various elements and biomolecules.

**Figure 1:**
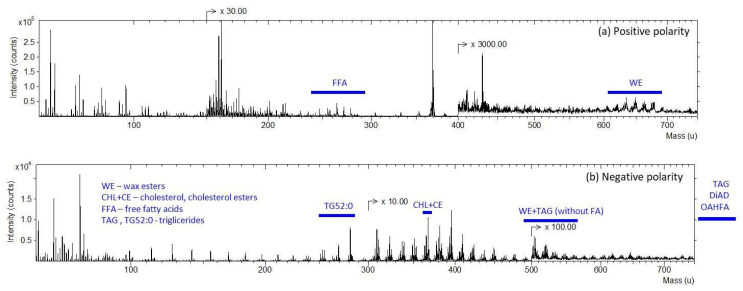
Positive (**a**) and negative (**b**) mass spectra for meibum collected from Participant D1.

In the first step of the analysis, the focus was placed on the main features of the spectra. Figure 1 displays TOF-SIMS spectra measured in both positive (Figure 1a) and negative (Figure 1b) polarities over a mass range of 50–750 u. The spectra reveal signals corresponding to various compound groups, with distinct peaks indicative of the compounds that constitute the composition of meibum. Among these, wax esters, cholesterol, free fatty acids, and triglycerides were specifically highlighted, allowing for the visual identification of their respective mass-to-charge ratios (*m*/*z*). The studies were conducted over a mass range of up to 750 u, which limited the detection of characteristic signals from compounds such as DiAD (diisopropylazodicarboxylate) and QAHFA (O-acyl hydroxy fatty acids), whose peak masses exceed this range. However, their identification remains feasible with an extended mass range, as the spectrometer used in this study allows for the analysis of spectra up to 20 ku.

**Figure 2:**
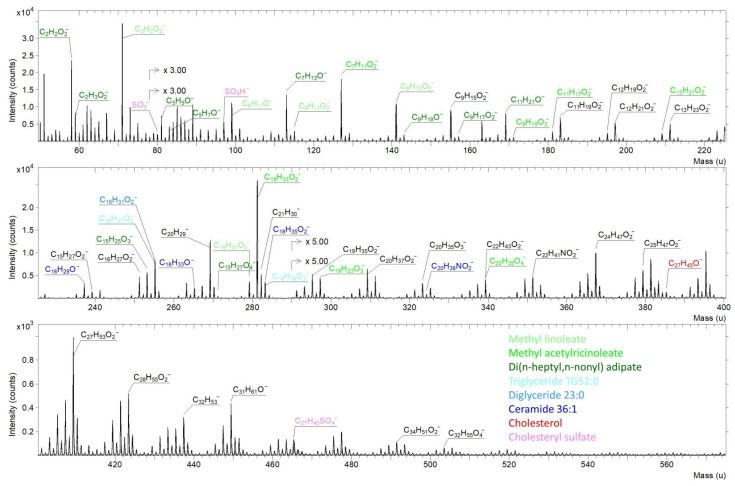
TOF-SIMS negative-ion spectra of meibum collected from Participant D1 with labeled characteristic peaks for selected detected compounds.

On the basis of the positive and negative mass spectra analyses of examined meibum samples, elemental and molecular ions were identified, allowing for the presence of particular biomolecules to be determined (see Figure 2—in this, only negative spectra are presented). The identification of ion signals from TOF-SIMS spectra enabled the detection of elements such as H, C, Na, Si, K, Cl, and O, as well as trace elements including P, F, and S. Subsequent analysis concentrated on signals enabling the identification of distinct groups of biomolecules; the built-in SurfaceLab database was used for the identification of compounds.

The presence of distinct biomolecular classes in meibum was confirmed by TOF-SIMS, with characteristic peaks identified in both positive and negative ion modes (see Figure 2). Free sterols, such as cholesterol, were observed at *m*/*z* 385.34 u (C_27_H_45_O^−^), while cholesteryl sulfate produced ions at *m*/*z* 97.00 u (SO_3_^−^) and *m*/*z* 96.96 u (HSO_4_^−^). Ceramide 36:1 was detected through multiple characteristic ions, including *m*/*z* 225.22 u (C_16_H_29_O^−^), *m*/*z* 257.26 u (C_18_H_33_O^−^), *m*/*z* 279.27 u (C_18_H_35_O_2_^−^), and *m*/*z* 328.29 u (C_20_H_38_NO_2_^−^), while diacylglycerols such as diglyceride 32:0 were identified at *m*/*z* 255.24 u (C_16_H_31_O_2_^−^). Triglycerides, represented by TG52:0, were similarly detected through representative fragment ions, including *m*/*z* 255.24 u (C_16_H_31_O_2_^−^) and *m*/*z* 281.27 u (C_18_H_35_O_2_^−^). Wax esters, encompassing methyl linoleate, methyl acetylricinoleate, and Di(n-heptyl, n-nonyl) adipate, exhibited characteristic ions such as *m*/*z* 113.08 u (C_7_H_11_O_2_^−^), 197.16 u (C_13_H_21_O_2_^−^), and 281.27 u (C_18_H_33_O_2_^−^) for methyl acetylrinoleate, and *m*/*z* 115.03 u (C_6_H_11_O_2_^−^), 125.09 u (C_9_H_17_O_2_^−^), 241.19 u (C_15_H_25_O_3_^−^), and 239.19 u (C_15_H_27_O_2_^−^) for Di(n-heptyl, n-nonyl) adipate. Monoglycerides were identified based on ions corresponding to hydrocarbon fragments and the acidic residue CH_3_CH_2_COO^+^ (*m*/*z* 59.01 u). Peaks corresponding to amino acid-related ions were detected at low masses, including NH_4_^+^ (*m*/*z* 18.04 u), CH_4_N^+^ (*m*/*z* 30.03 u), and CH_3_O^+^ (*m*/*z* 31.02 u); however, the absence of higher-mass peaks prevents the definitive confirmation of any specific amino acids within the examined samples. The compound assignments were confirmed using complementary data from both ionization modes, with characteristic peaks for each molecule observed in both positive and negative spectra.

Overall, the detected lipid profile confirms the known composition of meibum, dominated by barrier-forming wax esters and cholesterol [21,22]. The presence of polar lipids such as cholesteryl sulfate highlights their potential role as surfactants for lipid layer organization [21], while diacylglycerols and triglycerides may serve as biosynthetic intermediates [45,46]. Notably, altered levels of the latter can also be associated with biomarkers for gland dysfunction and other systemic diseases [6,7,8,9,10]. Key meibum components relevant for diagnostic differentiation in MGD include elevated linoleic acid, which correlates with clinical signs such as telangiectasia and gland plugging [14], and an altered cholesteryl-ester-to-wax-ester ratio indicative of disease severity [47]. A broader lipidomic pattern, characterized by decreased triacylglycerols, wax esters, and OAHFAs, alongside increased cholesteryl esters and phospholipids, may further distinguish MGD [45,47], particularly in systemic conditions such as type 2 diabetes [16]. These molecular insights could support the development of targeted therapeutic strategies aimed at normalizing the CE/WE ratio, restoring OAHFA levels to stabilize the tear film, and reducing pro-inflammatory lipid species. Current therapies for MGD are increasingly targeted at correcting specific pathological biomarkers found in meibum. For instance, elevated free fatty acids and inflammation are addressed with tetracycline antibiotics, which reduce these lipid-breakdown products [48,49]. Similarly, thermal pulsation devices aim to correct the abnormal lipid conformation and rigidity caused by an altered ratio of cholesterol esters to wax esters [50]. Furthermore, new therapeutic strategies, such as aldehyde traps, are being developed to counteract the pro-inflammatory aldehydes resulting from lipid peroxidation [47].

### 2.2. Inter-Individual Variability in Meibum Composition

#### 2.2.1. Detailed Spectral Analysis of Representative Samples

**Figure 3:**
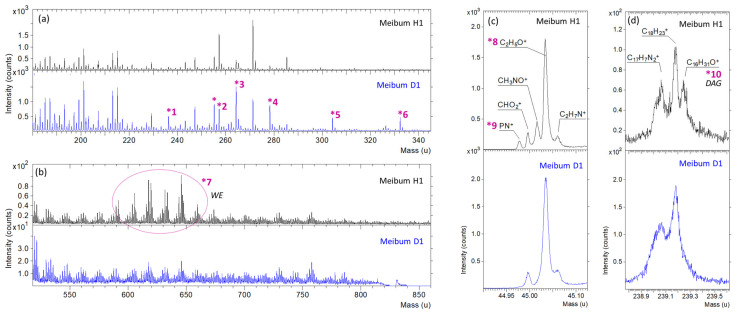
TOF-SIMS spectra of meibum collected from Participants H1 (black) and D1 (blue): (**a**) wide-mass-range spectra obtained in positive polarity; (**b**) wide-mass-range spectra obtained in negative polarity; (**c**,**d**) narrow-mass-range spectra. The peaks marked with asterisks indicate ions that clearly differentiate between H1 and D1 meibum. In the comparative plots, individual normalized intensity scales were applied to enhance the visibility of characteristic features.

A comparative analysis of the spectral fragments was conducted to assess the feasibility of differentiating meibum samples from various participants using this technique, and to identify the specific compound or molecular fragment responsible for distinguishing the samples. Two representative samples were selected for detailed comparison to illustrate the differences and highlight the compounds contributing to inter-individual variability (Figure 3a–d). The analysis disclosed subtle variations in the spectra of meibum samples collected from different individuals (Figure 3a–d). These variations in meibum composition can be categorized into two general types: (i) changes in the ratio of individual ions, as indicated by features *1–*4,*7 in Figure 3a,b (suggestive of potential lipid disorder), and (ii) the emergence of additional ions and, consequently, chemical compounds, as shown by features *5, *6,* 8–*10 (potential indicators of disease).

In the first variation type (Figure 3a,b), which highlights differences in the intensities of individual peaks, several notable variations were observed. The most significant differences (features *1–*7) were observed in the following ions: (*1) a peak at mass 236.27 u, identified as C_16_H_28_O^+^, likely originating from saturated or monounsaturated fatty acids (palmitic acid, palmitoleic acid) or their derivatives; (*2) a peak at mass 257.27 u, assigned to C_16_H_33_O_2_^+^, likely derived from palmitic acid or related saturated fatty acids, possibly in esterified forms like monoacylglycerides; (*3) a peak at 288.21 u, identified as C_15_H_27_O^+^, which may originate from arachidic or stearic acid, hydroxy fatty acids or oxidized unsaturated fatty acids; (*4) a peak at 264.32 u, assigned to C_18_H_32_O^+^, likely from phospholipids, glycerides, or eicosatrienoic acid; (*5) a peak at 304.29 u, identified as C_20_H_38_^+^, possibly from a saturated hydrocarbon chain in complex lipids like waxes or triglycerides; and (*6) a peak at 332.35 u, associated with C_24_H_44_^+^, likely a fragment of a long-chain unsaturated hydrocarbon from wax esters, cholesteryl esters or (O-acyl)-ω-hydroxy fatty acids. The increased intensity of ions derived from saturated and monounsaturated fatty acids (*1, *2, *6) could indicate a shift in lipid synthesis towards a more saturated composition, which is often associated with increased meibum viscosity and gland obstruction in MGD. For the group of peaks (*7), the most prominent peaks correspond to C_42_H_65_O^−^, C_32_H_61_O_11_, C_39_H_67_O_6_^−^, and C_40_H_69_O_6_^−^; these ions are likely derived from complex lipids such as triacylglycerols (e.g., TG 54:6) or glycolipids, known to be involved in lipid metabolism and signaling. The prevalence of these fragments suggests variations in fatty acid saturation and complex lipid metabolism between the samples.

In the second type of variation in chemical composition (see Figure 3c,d), associated with the presence of additional peaks in the examined meibum samples, several differences were observed. In the meibum collected from H1, a few notable additional peaks were detected: (*8) a peak at mass 45.02 u, identified as CH_3_NO^+^, could indicate the presence of amino acids (for example, serine might produce CH_3_NO^+^ fragments upon ionization); however, such a peak can also be attributed to ceramides or phospholipids, (*9) a peak at mass 44.98 u (PN^+^), a characteristic fragment associated primarily with phospholipids, and (*10) a peak at mass 329.24 u, identified as C_16_H_31_O^+^, a classic diacylglycerol-type fragment consistent with the presence of triglycerides. The unique presence of the phospholipid-derived PN^+^ fragment and the distinct DG-type ion in Participant D1 points to a fundamentally different lipid composition, potentially indicative of specific metabolic or pathogenic states.

Observed differences in peak intensities and the presence of unique features suggest significant variations in lipid metabolism, saturation states, and potential oxidative processes between the examined meibum samples. The prevalence of fragments associated with fatty acids (e.g., *1, *2, *3, *6), complex glycerides (*7, *10), and phospholipids (*4, *9) in the comparative analysis points to an imbalance in the relative abundances of meibum’s core lipid classes between meibum collected from different participants, suggesting underlying alterations in their synthetic pathways. In the Supplementary Materials, Table S1 summarizes the normalized areas under the peaks for the full group of analyzed meibum samples used in the statistical evaluation.

#### 2.2.2. Principal Component Analysis of the Expanded Cohort

To provide a statistical foundation for our findings, the complete dataset of ten samples was subjected to a multi-tiered analytical approach. First, we performed multivariate tests on positive ions to ascertain the overall compositional differences between the groups. The PERMANOVA test resulted in a test statistic of 2.873, with a *p*-value of 0.042, showing that the two groups differ significantly. The permutational analysis ofmultivariate dispersions (PERMDISP) test with a *p*-value of 0.057 showed that there were no significant differences in the group dispersion, so it is unlikely that the previous result was affected by this. The MMD test further confirms this result, showing a significant difference between the groups, with a *p*-value of 0.009.

Next, to identify the specific spectral features driving this separation, we conducted a detailed, fragment-level analysis of the positive ion data. The group separation analysis based on individual ions (Table 1, derived from Table S1, Supplementary Materials) shows, for each analyzed ion, the *p*-value of the Welch’s *t*-test (column 2) and the effect size obtained using Cohen’s d (column 3). We notice that the Welch’s test *p*-values are rather large; however, this is to be expected in tests with rather small sample sizes. We thus based our further analysis on the group separation effect sizes. The effect sizes of seven of the analyzed ions can be considered large, with absolute values exceeding 0.8, and with the C_2_H_5_O^+^, CHO_2_^+^, C_18_H_23_^+^, and C_2_H_7_N^+^ ions reaching very high effect sizes (over 1.2) [51].

This targeted statistical comparison confirms the presence of disease-specific molecular alterations at the level of individual spectral fragments.

The consistency of these findings was further validated by a supervised partial least squares–discriminant analysis (PLS-DA)—analyzing the results of the PLS-DA analysis in Figure S1a (Supplementary Materials), a clear separation between the groups is visible. Additionally, the absolute values of loadings in Figure S1b (Supplementary Materials), which show the importance of each ion in group separation, have the same ordering as the effect sizes in Table 1, indicating consistency between the two analyses.

**Figure 4:**
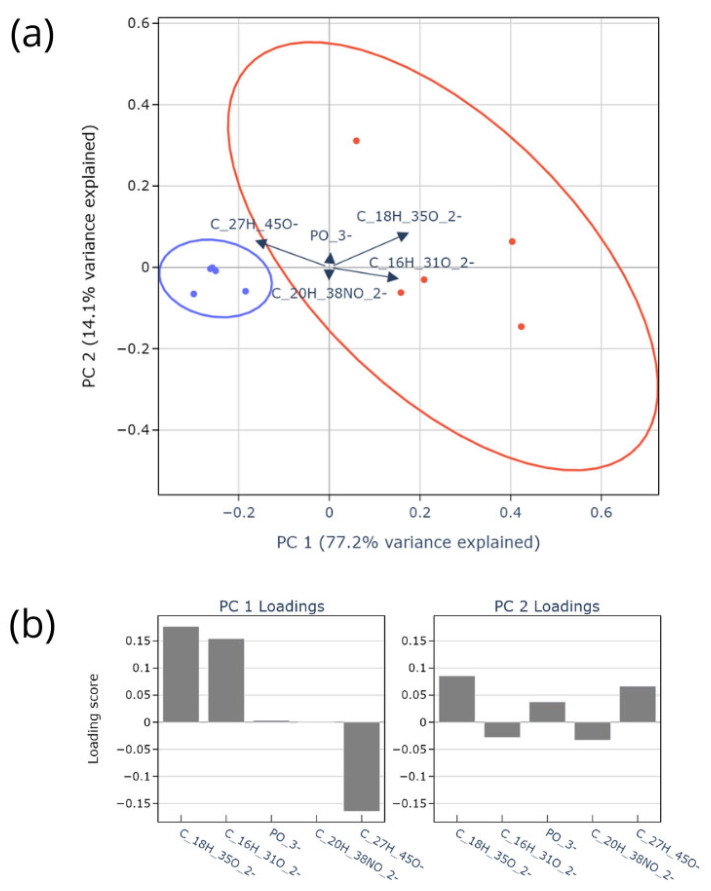
PCA of the data: (**a**) data points projected to the first two principal components of the PCA. The arrows present the directions of original features used in PCA with relation to the principal components. The colors represent the two data clusters and the corresponding confidence ellipses. (**b**) Loading scores of the original features for the two principal components.

Finally, to visualize the overarching lipidomic patterns and relate them to biologically defined lipid classes, we performed a PCA on a focused set of five diagnostic anions from the negative ion spectra C_18_H_35_O_2_^−^, C_16_H_31_O_2_^−^, C_20_H_38_NO_2_^−^, C_27_H_45_O^−^, and PO_3_^−^ (Figure 4a,b). These ions represent key classes of meibum lipids: unsaturated fatty acids critical for fluidity (C_16_H_31_O_2_^−^, C_18_H_35_O_2_^−^) [24], cholesterol (C_27_H_45_O^−^), which regulates lipid layer organization [25], a polar nitrogen-containing fragment (C_20_H_38_NO_2_^−^), and a general phosphate fragment (PO_3_^−^), potentially derived from trace phospholipids [52]. The first two principal components of the analysis explain most of the variance in the original data (Figure 4a)—77.2% and 14.1% of the variance, respectively. There are two distinct groups of data points which are split nearly exclusively along the first PC, with one group receiving positive values and the other negative values. This separation was confirmed by K-Means clustering, which assigned samples to a blue and a red group corresponding precisely to healthy controls and patients with MGD, respectively.

The loadings plot (Figure 4b) quantifies the contribution of each ion to this separation. PC1, which captures the between-group variance, is defined by strong positive loadings from the unsaturated fatty acids C_18_H_35_O_2_^−^ and C_16_H_31_O_2_^−^ and a strong negative loading from cholesterol (C_27_H_45_O^−^). Consequently, the MGD (red) cluster is characterized by a lipid profile that is enriched in these unsaturated fatty acids, while the healthy (blue) cluster is associated with higher relative levels of cholesterol. The ions C_20_H_38_NO_2_^−^ and PO_3_^−^ had negligible loadings on PC1, indicating they do not drive the primary group separation.

The second principal component (PC2) captures within-group variability. This is primarily influenced by positive loadings from C_18_H_35_O_2_^−^ and C_27_H_45_O^−^, revealing that individual variability in oleic acid and cholesterol levels is correlated within each clinical group, potentially reflecting personal physiological or metabolic factors independent of disease status. The contributions of C_20_H_38_NO_2_^−^ and PO_3_^−^ to PC2 are present but comparatively minor.

An interesting observation emerges when comparing our PCA results with studies using other analytical techniques. While LC-MS analyses often report decreased saturated fatty acids (C16:0, C18:0) in MGD meibum [53], and Raman spectroscopy similarly indicates a reduction in unsaturated lipid content [54], our TOF-SIMS data reveal a distinct trend. We observed a relative increase in monounsaturated species C_18_H_35_O_2_^−^ (C18:1) and C_16_H_31_O_2_^−^ (C16:1) in the disease group, as evidenced by their strong positive loadings on PC1, paired with a concurrent relative decrease in free cholesterol (C_27_H_45_O^−^), indicated by its strong negative loading (Figure 4b). The decrease in cholesterol in MGD reported in the literature [24] aligns with our finding. However, reports of increased unsaturated fatty acids are typically specific to very long-chain species (C25–C30) [55], indicating a complex, non-uniform shift in the unsaturated lipid pool. Our surface-sensitive TOF-SIMS profile, highlighting C18:1 and C16:1, may therefore capture a specific facet of this reorganization—suggesting a relative surface enrichment of these more fluidizing species. This divergence likely reflects the distinct methodological perspectives: TOF-SIMS provides a semi-quantitative, surface-sensitive profile of lipid organization, while LC-MS measures the bulk, quantitative composition and Raman spectroscopy provides volumetric compositional information. The opposing loadings on PC1 suggest a fundamental shift in the meibum’s physicochemical state in MGD, where surface-accessible monounsaturated fatty acids become more prominent, and cholesterol is depleted. This may indicate a compensatory biochemical response, such as increased desaturation to counteract increased viscosity, or a surface-specific reorganization of the lipid layer.

## 3. Materials and Methods

### 3.1. Participant Selection and Clinical Characteristics

Ten participants were included in the study, all White females, comprising five healthy individuals and five with clinically confirmed meibomian gland dysfunction (MGD), aged 28 to 46 years with comparable age distribution between groups. Including only females minimized biological variability related to sex, allowing for a preliminary assessment of TOF-SIMS’s ability to detect differences in meibum composition. Due to the small sample size, stratification by menstrual cycle phase was not feasible. However, future studies with larger cohorts should consider this factor given its potential impact on meibum composition [56,57]. Table 2 summarizes the key demographic characteristics of the cohort, including participant age and MGD score and severity according to TFOS criteria.

Participants were selected from a pool of female volunteers among university staff based on eyelid and meibum characteristics, following a comprehensive ophthalmic examination performed by an experienced ophthalmologist using a slit-lamp biomicroscope. MGD assessment was based on standard diagnostic criteria, including inspection of the eyelid margins, evaluation of the ocular surface, and characterization of meibum expressibility and quality. Meibomian gland function was evaluated as follows: meibum expressibility on a 0–3 scale (based on the number of glands in the lower eyelid from which secretion could be obtained under mild digital pressure) and meibum quality on a 0–3 scale (from clear fluid [0] to opaque, toothpaste-like material [3]) [10].

Healthy participants exhibited normal eyelid margins, full meibum expressibility (0), and clear secretions (0). Participants with MGD showed reduced expressibility (1–2) and impaired secretion quality (1–2), corresponding to mild to moderate MGD.

In the subsequent analyses, TOF-SIMS spectra from two representative participants, one healthy (H1) and one with MGD (D1), were presented to illustrate clear differences in meibum composition. Collective PCA was performed on all ten samples, allowing for assessment of whether the method could distinguish healthy and MGD samples at the group level based on the five main biomarkers.

### 3.2. Collection and Processing of Human Meibum

After local drip anesthesia of the conjunctival sac with proxymetacaine hydrochloride 0.5% eye drops (Alcaine, Alcon Polska, Warsaw, Poland), the edges of the upper and lower eyelids and eyelashes were washed with sterile saline. Then, the margins of the upper and lower eyelids were pressed with a sterile round glass rod with spatula end (Conbest, Kraków, Poland) in the area of the meibomian glands and a small amount of about 1mg of secretion (meibum) was collected. During meibum collection, the eyelid was gently everted to minimize contamination from tear film components. The collected sample was placed on a glass slide, covered with a coverslip, labeled, and stored in a suitable container at 8 °C. Within six hours of collection, an analysis of the chemical composition of the sample was carried out using the TOF-SIMS 5 spectrometer located in the Surface Physics Laboratory.

**Figure 5:**
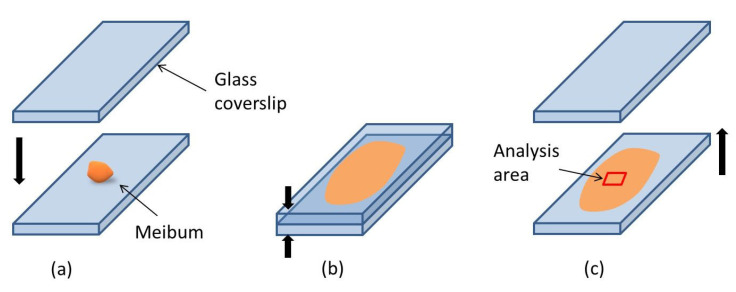
Schematic of the preparation of the meibum for TOF-SIMS examination. (**a**) Placement of the meibum sample on a glass coverslip. (**b**) Spreading and flattening of the sample by covering it with a second coverslip and applying gentle pressure. (**c**) After separation of the coverslips, a uniform meibum layer remains on one slide; the red square indicates the selected TOF-SIMS analysis area. The arrows illustrate the manipulation of the coverslips.

The meibum, once collected, was sandwiched between two coverslips, and then, through applying slight pressure by hand, the collected meibum was flattened (see Figure 5a,b). This coverslip-assisted meibum preparation procedure resulted in a flat surface of the collected material and increased the area for further analysis (Figure 5c). Moreover, the placement of meibum between the slides protected the material from unwanted drying, oxidation, and the appearance of contaminants on the material’s surface.

**Figure 6:**
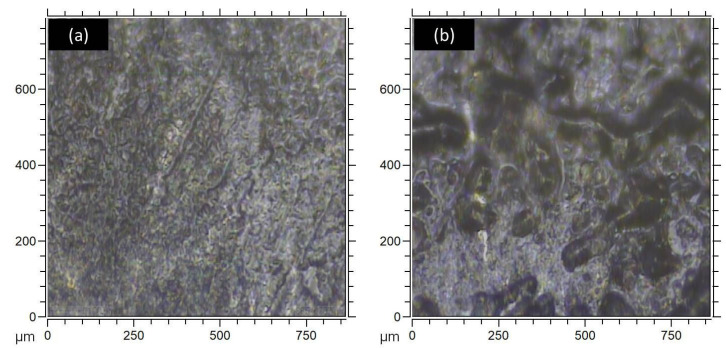
Microscopic image of examined meibum samples (**a**) collected from Participant H1 and (**b**) collected from Participant D1. A 500 × 500 µm area centered in the middle of the image was selected for analysis. The meibum forms a uniform, continuous layer across the surface of the glass slide. Variations in surface morphology may result from the method of cover slip separation and biological differences in meibum composition.

Collected samples were individually packaged and stored at 8 °C to prevent, or at least minimalize, chemical degradation of the meibum by slowing enzymatic activity, hydrolysis, or oxidation. The measurements of the chemical composition were executed within 24 h after the collection of the material. Prior to measurements, the top coverslip was carefully removed to expose the sample surface (see Figure 5c). Both coverslips were then mounted on the sample holder and later placed in the analysis chamber of TOF-SIMS spectrometer. Although the ultra-high-vacuum (UHV) conditions required for TOF-SIMS are not ideal for lipids, potentially causing loss of volatiles, structural changes, or altered composition, the measurements were carried out with caution, with potential evaporation or visible changes monitored using the spectrometer’s built-in microcamera. The pump-down time from atmospheric pressure to high vacuum (10^−7^ mbar) for the sample holder containing mounted samples was consistently maintained within 30 min. After placing the samples in the measuring chamber, the vacuum stabilized at approximately 2 × 10^−8^ mbar. Overall, the vacuum did not alter the structure of the thin meibum layer; the layer remained continuous in the tested sample (see Figure 6a,b). Microscopic imaging confirmed that the meibum layer uniformly and continuously coated the coverslip, with no detectable uncoated regions. The differences in image morphology are likely attributable to variations in technical handling during sample preparation, particularly the method of coverslip removal (whether it is slid off horizontally or peeled vertically), as well as to biological variation in meibum composition between participants.

### 3.3. Time of Flight Secondary Ion Mass Spectrometry

Time-of-flight secondary ion mass spectrometry (TOF-SIMS) is an analytical technique for chemical analysis that involves directing a pulsed beam of primary ions onto a sample surface, leading to the generation of secondary ions through a sputtering process. The analysis of these secondary ions offers insights into the elemental and molecular species that exist on the surface of characterized material [58]. By focusing the beam to sub-micrometer dimensions, the technique enables the analysis of features within the range of 1 µm to 500 µm. The depth of analysis of the TOF-SIMS technique is very shallow; it is expected that secondary ions can be excited from a depth of 1–2 nm, which is why the TOF-SIMS technique is listed as one of the surface-sensitive techniques. Combining its detection sensitivity in the part-per-million (ppm) range with its high surface sensitivity and ability to analyze submicron areas, TOF-SIMS is an ideal tool for the chemical characterization of relatively small samples or samples that inherently exist in limited quantities and can be prepared as thin films.

TOF–SIMS measurements were carried out with the use of a TOF–SIMS 5 (ION-TOF GmbH, Munster, Germany) reflection-type spectrometer, equipped with bismuth liquid metal ion gun, Bi_3_^+^ of energy of 30 keV, and a current of about 0.5 pA. Positive and negative secondary ion spectra were collected by rastering the ion beam across predetermined 500 × 500 µm areas. The acquisition of both positive and negative ion spectra is a standard practice in TOF-SIMS, as each polarity selectively enhances the detection of different elemental and molecular species, thereby providing a more comprehensive analysis.The spectra were collected in the range 1–1000 u for both polarities. The measurement time of a single sample did not exceed the static conditions of the measurement (ion dose per square centimeter below 10^12^) to avoid sample damage. Each sample was analyzed under consistent bismuth ion gun parameters, measurement duration, and analyzer settings. The flood gun was used in order to compensate surface-charging. The analysis was carried out using SurfaceLab 6 software (IONTOF GmbH, Munster, Germany).

### 3.4. Statistical Analysis

We used several statistical tests to determine the degree to which the data acquired using the TOF-SIMS technique enables us to distinguish healthy and diseased meibum samples. Due to the small sample size of our data, we used permutation tests, which resample the data to obtain the test statistic. We first performed a non-parametric permutational multivariate analysis of variance (PERMANOVA) [59] test to determine whether the locations and dispersions of healthy and diseased groups of samples differ significantly. We then used permutational analysis of multivariate dispersions (PERMDISP) to test whether differences in group dispersions might affect the result of PERMANOVA. We also used the non-parametric kernelized maximum mean discrepancy (MMD) test [60] to further test the difference in the chemical compositions of the two groups. To measure the difference in the samples caused by each of the measured ions, we performed a Welch’s *t*-test [61]. The *p*-values for each ion were then corrected using the Benjamini–Hochberg multiple testing correction. Furthermore, for each ion, we calculated Cohen’s d [62] effect size measure. Finally, to visualize the difference between the groups along the direction of the greatest group separation, we used partial least-squares discriminant analysis (PLS-DA).

To aid in analyzing and visualizing the data acquired using the TOF-SIMS technique, a dimensionality reduction using PCA [63] was conducted on the samples. The PCA outputs the data points projected along principal components (PC) of the data, or the consecutive directions in the original feature space, which capture the highest variance in the data distribution. The data points projected to the first two principal components of the PCA were visualized in the form of a scatterplot. The directions of the original features with respect to the principal components were overlayed on the scatterplot. The loading scores of the original features with respect to the principal components were analyzed as well. These values can range from −1 to 1 and they indicate the impact a feature has on the principal component. A positive loading score means that higher values of the feature contribute positively to the value of the PC, while negative loading scores signify that lower values of the original feature will increase the values of the PC.

Subsequently, the data points were clustered into two groups using a nonparametric, unsupervised clustering method, K-Means clustering [64]. The clustering divides the data into groups based on the proximity between the data points. Based on the clusterings, the distribuitons of the data points can then be estimated. A bivariate normal distribution of the points is assumed and the joint uncertainty of the data is visualized in the form of confidence ellipses at three standard deviations from the means of the clusters.

We used the following Python packages for statistic calculations and visualizations: scikit-bio version 0.7.1 was used for calculating PERMDISP, PERMANOVA and MMD; SciPy version 1.17.0 was used for calculating the Welch’s *t*-test, multiple testing correction, and Cohen’s d; scikit-learn version 1.8.0 was used for PLS-DA, PCA, and K-Means clustering. The visualizations were generated using Plotly version 6.5.2.

## 4. Conclusions

The study aimed to verify the applicability of the TOF-SIMS technique in meibum research. From a technical point of view, the preparation of meibum for the study proposed in this paper allowed the sample to be placed safely in the spectrometer and measurements to be carried out under ultra-high-vacuum conditions. The amount of material collected was sufficient to provide a continuous coating on the coverslip, allowing for the easy selection of the area to be analyzed. The results obtained from analyses of ten samples collected from different participants provided information on the chemical composition of meibum, confirming the lipid nature of the material under investigation. In the analysis of meibum, significant variations in peak intensities revealed the presence of various lipid-related compounds: saturated and monounsaturated fatty acids (e.g., palmitic and palmitoleic acids), hydroxy and oxidized fatty acids, phospholipids, and complex lipids such as glycerides, glycolipids, and waxes. Crucially, multivariate statistical analysis—PCA of the TOF-SIMS data—revealed a distinct lipidomic signature that clearly differentiated samples from healthy participants and those with MGD. This signature was primarily driven by an elevated ratio of specific monounsaturated fatty acids to cholesterol in the MGD group, demonstrating the technique’s sensitivity to disease-associated biochemical changes.

The application of TOF-SIMS studies is valuable due to its ability to detect elemental or molecular signals from compounds present in trace amounts owing to its very low detection limits. The ability to detect a wide range of compounds and, as shown, to statistically differentiate pathological states based on lipid profiles within a relatively short experimental timeframe, further underscores the utility of the TOF-SIMS technique in meibum research.

This study has two main limitations. The sample size was small, with only five healthy participants and five patients with clinically confirmed MGD, due to the pilot nature of the study and the novel use of TOF-SIMS for meibum analysis. Only female volunteers were included to reduce biological variability. However, because of the small sample size, menstrual cycle phase was not taken into account in order to avoid over-stratifying the groups, although hormonal changes may affect meibum composition. Future studies with larger and more diverse groups should include both sexes and consider hormonal status and menstrual cycle phase to better understand meibum variability.

Nevertheless, preliminary results obtained using a newly implemented protocol for analyzing meibum composition using TOF-SIMS, tested on participants with differing degrees of gland dysfunction, further support its potential for more detailed and non-invasive lipid profiling. If validated in broader studies, this approach could contribute to the earlier detection and more personalized management of Meibomian gland dysfunction.

## Figures and Tables

**Table 1:** Results of the Welch’s *t*-test for individual ions after multiple testing correction, along with their corresponding Cohen’s d effect sizes.

Ion	*p*-Value	Effect Size
C_2_H_5_O^+^	0.170690	2.659686
CHO_2_^+^	0.175155	1.803090
C_18_H_23_^+^	0.175155	−1.737394
C_2_H_7_N^+^	0.175155	1.700526
C_16_H_33_O_2_^+^	0.328902	1.192288
C_16_H_31_O^+^	0.328902	1.190528
C_17_H_7_N_2_^+^	0.351117	−0.976241
PN^+^	0.484294	0.790003
C_15_H_27_O^+^	0.632202	0.593808
CH_3_NO^+^	0.703237	0.457285
C_18_H_32_O^+^	0.703237	0.449847
C_16_H_28_O^+^	0.776160	0.320658
C_21_H_38_N^+^	0.776160	0.280560
C_24_H_44_^+^	0.822646	0.174471
C_20_H_38_^+^	0.822646	0.149186

**Table 2:** Key demographic characteristics of the study cohort of female volunteers, including age and MGD severity based on TFOS criteria. Meibum expressibility and quality were scored on a 0–3 scale.

Subject ID	Group	Meibum Expressibility (0–3)	Meibum Quality (0–3)	Age (Years)	MGD Severity (TFOS)
H1	Healthy	0	0	43	None
H2	Healthy	0	0	32	None
H3	Healthy	0	0	29	None
H4	Healthy	0	0	41	None
H5	Healthy	0	0	35	None
D1	MGD	1	1	46	Mild
D2	MGD	1	2	31	Mild-to-Moderate
D3	MGD	2	1	36	Mild-to-Moderate
D4	MGD	2	2	42	Moderate
D5	MGD	2	2	28	Moderate

## Data Availability

The data are not publicly available due to confidentiality and ethical considerations.

## Supplementary Materials

The following supporting information can be downloaded at: https://www.mdpi.com/article/10.3390/ijms27031590/s1. Table S1. Normalized to total intensity peak areas of characteristic positive ions for the complete cohort of meibum samples. Figure S1. PLS-DA analysis of the data: (a) Data points projected to the two PLS components. The colors represent the two data clusters and the corresponding confidence ellipses. (b) PLS loading scores showing the level of contribution of each ion to the group separation.

## References

[1] Jester J.V., Nicolaides N., Smith R.E. (1981). Meibomian Gland Studies: Histologic and Ultrastructural Investigations. Invest. Ophthalmol. Vis. Sci..

[2] Knop E., Knop N., Millar T., Obata H., Sullivan D.A. (2011). The International Workshop on Meibomian Gland Dysfunction: Report of the Subcommittee on Anatomy, Physiology, and Pathophysiology of the Meibomian Gland. Invest. Ophthalmol. Vis. Sci..

[3] Craig J.P., Tomlinson A. (1997). Importance of the Lipid Layer in Human Tear Film Stability and Evaporation. Optom. Vis. Sci..

[4] Bron A.J., Tiffany J.M., Gouveia S.M., Yokoi N., Voon L.W. (2004). Functional Aspects of the Tear Film Lipid Layer. Exp. Eye Res..

[5] Jones M.B., McElwain D.L., Fulford G.R., Collins M.J., Roberts A.P. (2006). The Effect of the Lipid Layer on Tear Film Behavior. Bull. Math. Biol..

[6] Willcox M.D.P., Argüeso P., Georgiev G.A., Holopainen J.M., Laurie G.W., Millar T.J., Papas E.B., Rolland J.P., Schmidt T.A., Stahl U. (2017). TFOS DEWS II Tear Film Report. Ocul. Surf..

[7] Mathers W.D. (1993). Ocular Evaporation in Meibomian Gland Dysfunction and Dry Eye. Ophthalmology.

[8] Shimazaki J., Sakata M., Tsubota K. (1995). Ocular Surface Changes and Discomfort in Patients with Meibomian Gland Dysfunction. Arch. Ophthalmol..

[9] Foulks G.N., Bron A.J. (2003). Meibomian Gland Dysfunction: A Clinical Scheme for Description, Diagnosis, Classification, and Grading. Ocul. Surf..

[10] Tomlinson A., Bron A.J., Korb D.R., Amano S., Paugh J.R., Pearce E.I., Yee R., Yokoi N., Arita R., Dogru M. (2011). The International Workshop on Meibomian Gland Dysfunction: Report of the Diagnosis Subcommittee. Invest. Ophthalmol. Vis. Sci..

[11] Nichols K.K., Foulks G.N., Bron A.J., Glasgow B.J., Dogru M., Tsubota K., Lemp M.A., Sullivan D.A. (2011). The International Workshop on Meibomian Gland Dysfunction: Executive Summary. Invest. Ophthalmol. Vis. Sci..

[12] Nguyen Pham K.T., Miyake T., Suzuki T., Kinoshita S., Hamada Y., Uehara H., Machida M., Nakajima T., Hasegawa E., Doi M. (2025). Identification of Meibomian Gland Testosterone Metabolites Produced by Tissue-Intrinsic Intracrine Deactivation Activity. iScience.

[13] Nguyen A., Naidoo K.K., Ajouz L., Xu X., Zhao C., Robinson M.R., Borchman D. (2024). Changes in Human Meibum Lipid Composition Related to the Presence and Severity of Meibomian Gland Dysfunction. J. Ocul. Pharmacol. Ther..

[14] Arita R., Mori N., Shirakawa R., Asai K., Imanaka T., Fukano Y., Nakamura M. (2016). Linoleic Acid Content of Human Meibum Is Associated with Telangiectasia and Plugging of Gland Orifices in Meibomian Gland Dysfunction. Exp. Eye Res..

[15] Ewurum A., Veligandla S.R., Swindle J.S., Clark J.D., Borchman D. (2021). A Spectroscopic Approach to Measuring Meibum Lipid Composition and Conformation in Donors with Sjӧgren’s Syndrome. Exp. Eye Res..

[16] Yang Q., Li B., Sheng M. (2021). Meibum Lipid Composition in Type 2 Diabetics with Dry Eye. Exp. Eye Res..

[17] Chen J., Panthi S. (2019). Lipidomic Analysis of Meibomian Gland Secretions from the Tree Shrew: Identification of Candidate Tear Lipids Critical for Reducing Evaporation. Chem. Phys. Lipids.

[18] Ou S., Jeyalatha M.V., Mao Y., Wang J., Chen C., Zhang M., Liu X., Liang M., Lin S., Wu Y. (2022). The Role of Ectodysplasin A on the Ocular Surface Homeostasis. Int. J. Mol. Sci..

[19] Liu Y., Butovich I.A., Garreis F., Zahn I., Scholz M., Gaffling S., Jabari S., Dietrich J., Paulsen F. (2024). Comparative Characterization of Human Meibomian Glands, Free Sebaceous Glands, and Hair-Associated Sebaceous Glands Based on Biomarkers, Analysis of Secretion Composition, and Gland Morphology. Int. J. Mol. Sci..

[20] Wang J., Kang H., Huang X., Liu Y., He Y., Jie Y. (2025). Identification of Microplastics in Human Tear Fluid and Meibum: Implications for Dry Eye Disease Pathogenesis. J. Hazard. Mater..

[21] Butovich I.A., Suzuki T., Wojtowicz J., Bhat N., Yuksel S. (2020). Comprehensive Profiling of Asian and Caucasian Meibomian Gland Secretions Reveals Similar Lipidomic Signatures Regardless of Ethnicity. Sci. Rep..

[22] Ewurum A., Ankem A., Georgiev G., Borchman D. (2021). A Spectroscopic Study of the Composition and Conformation of Cholesteryl and Wax Esters Purified from Meibum. Chem. Phys. Lipids.

[23] Butovich I.A. (2017). Meibomian Glands, Meibum, and Meibogenesis. Exp. Eye Res..

[24] Schnetler R., Gillan W.D.H., Koorsen G. (2013). Lipid Composition of Human Meibum. Afr. Vis. Eye Health.

[25] Butovich I.A. (2009). The Meibomian Puzzle: Combining Pieces Together. Prog. Retin. Eye Res..

[26] Butovich I.A. (2011). Lipidomics of Human Meibomian Gland Secretions: Chemistry, Biophysics, and Physiological Role of Meibomian Lipids. Prog. Lipid Res..

[27] Robosky L.C., Wade K., Woolson D., Baker J.D., Manning M.L., Gage D.A., Reily M.D. (2008). Quantitative Evaluation of Sebum Lipid Components with Nuclear Magnetic Resonance. J. Lipid Res..

[28] Borchman D., Ramasubramanian A. (2019). Human Meibum Chain Branching Variability with Age, Gender and Meibomian Gland Dysfunction. Ocul. Surf..

[29] Haworth K.M., Nichols J.J., Thangavelu M., Sinnott L.T., Nichols K.K. (2011). Examination of Human Meibum Collection and Extraction Techniques. Optom. Vis. Sci..

[30] Chen J. (2021). Mass Spectrometric Analysis of Meibomian Gland Lipids. Methods Mol. Biol..

[31] Garcia-Queiruga J., Pena-Verdeal H., Sabucedo-Villamarin B., Paz-Tarrio M., Guitian-Fernandez E., Garcia-Resua C., Yebra-Pimentel E., Giraldez M.J. (2024). Meibum Lipidomic Analysis in Evaporative Dry Eye Subjects. Int. J. Mol. Sci..

[32] Butovich I.A., Uchiyama E., McCulley J.P. (2007). Lipids of Human Meibum: Mass-Spectrometric Analysis and Structural Elucidation. J. Lipid Res..

[33] Shine W.E., McCulley J.P. (2004). Meibomianitis: Polar Lipid Abnormalities. Cornea.

[34] Shine W.E., McCulley J.P. (2003). Polar Lipids in Human Meibomian Gland Secretions. Curr. Eye Res..

[35] Oshima Y., Sato H., Zaghloul A., Foulks G.N., Yappert M.C., Borchman D. (2009). Characterization of Human Meibum Lipid Using Raman Spectroscopy. Curr. Eye Res..

[36] Butovich I.A. (2008). On the Lipid Composition of Human Meibum and Tears: Comparative Analysis of Nonpolar Lipids. Invest. Ophthalmol. Vis. Sci..

[37] Gulin A.A., Pavlyukov M.S., Gusev S.A., Malakhova Y.N., Buzin A.I., Chvalun S.N., Aldarov K.G., Klinov D.V., Gularyan S.K., Nadtochenko V.A. (2017). Applicability of TOF-SIMS for the Assessment of Lipid Composition of Cell Membrane Structures. Biochem. Suppl. Ser. A Membr. Cell Biol..

[38] Dowlatshahi Pour M., Jennische E., Lange S., Ewing A.G., Malmberg P. (2016). Food-Induced Changes of Lipids in Rat Neuronal Tissue Visualized by ToF-SIMS Imaging. Sci. Rep..

[39] Shi X., Li X., Li Q., Qi C., Xia M., Wang Z., Chen Y., Zhou Z., Wang Z., Abliz Z. (2024). A ToF-SIMS Methodology for Analyzing Inter-Tissue Lipid Distribution Variations and Intra-Tissue Multilevel Mass Spectrometry Imaging within a Single Rat. Microchem. J..

[40] Park J.W., Jeong H., Kang B. (2015). Multi-Dimensional TOF-SIMS Analysis for Effective Profiling of Disease-Related Ions from the Tissue Surface. Sci. Rep..

[41] Denbigh J.L., Lockyer N.P. (2015). ToF-SIMS as a Tool for Profiling Lipids in Cancer and Other Diseases. Mater. Sci. Technol..

[42] Touboul D., Roy S., Germain D.P., Chaminade P., Brunelle A., Laprévote O. (2007). MALDI-TOF and Cluster-TOF-SIMS Imaging of Fabry Disease Biomarkers. Int. J. Mass Spectrom..

[43] Almstrand A.C., Josefson M., Bredberg A., Lausmaa J., Sjövall P., Larsson P., Olin A.C. (2012). TOF-SIMS Analysis of Exhaled Particles from Patients with Asthma and Healthy Controls. Eur. Respir. J..

[44] Neittaanmäki N., Zaar O., Sjögren K., Kelly C., Nilsson D., Katsarelias D., Bagge R.O., Paoli J., Fletcher J.S., Dimovska K. (2024). ToF-SIMS Imaging Reveals Changes in Tumor Cell Lipids during Metastatic Progression of Melanoma. Pigment. Cell Melanoma Res..

[45] Zhao W., Yang J., Liao Y., Yang B., Lin S., Liu R., Liang L. (2023). Alteration of Meibum Lipidomics Profiling in Patients With Chronic Ocular Graft-Versus-Host Disease. Invest. Ophthalmol. Vis. Sci..

[46] Suzuki T., Kitazawa K., Cho Y., Yoshida M., Okumura T., Sato A., Kinoshita S. (2022). Alteration in Meibum Lipid Composition and Subjective Symptoms Due to Aging and Meibomian Gland Dysfunction. Ocul. Surf..

[47] Nagar S., Ajouz L., Nichols K.K., Kumar S., Zhao C., Naidoo K.K., Robinson M.R., Borchman D. (2023). Relationship between human meibum lipid composition and the severity of meibomian gland dysfunction: A spectroscopic analysis. Invest. Ophthalmol. Vis. Sci..

[48] Mathebula S.D. (2022). Latest Developments on Meibomian Gland Dysfunction: Diagnosis, Treatment and Management. Afr. Vis. Eye Heal..

[49] Sabeti S., Kheirkhah A., Yin J., Dana R. (2020). Management of Meibomian Gland Dysfunction: A Review. Surv. Ophthalmol..

[50] Tao J.P., Shen J.F., Aakalu V.K., Foster J.A., Freitag S.K., McCulley T.J., Vagefi M.R., Kim S.J., Wladis E.J. (2023). Thermal Pulsation in the Management of Meibomian Gland Dysfunction and Dry Eye: A Report by the American Academy of Ophthalmology. Ophthalmology.

[51] Sawilowsky S.S. (2009). New Effect Size Rules of Thumb. J. Mod. Appl. Stat. Methods.

[52] Lam S.M., Tong L., Yong S.S., Li B., Chaurasia S.S., Shui G., Wenk M.R. (2011). Meibum Lipid Composition in Asians with Dry Eye Disease. PLoS ONE.

[53] Wojtowicz J.C., Butovich I.A., McCulley J.P. (2009). Historical Brief on Composition of Human Meibum Lipids. Ocul. Surf..

[54] Ou S., Zhang L., Wu Y., Yang D., Jiang N., Mao T., Zheng X., Gu H., Zhang L. (2025). Alterations in the Composition of Meibomian Gland Secretions in Patients with Meibomian Gland Dysfunction Based on Raman Spectroscopy. Front. Med..

[55] Arita R., Mori N., Shirakawa R., Asai K., Imanaka T., Fukano Y., Nakamura M., Amano S. (2015). Meibum Color and Free Fatty Acid Composition in Patients With Meibomian Gland Dysfunction. Invest. Ophthalmol. Vis. Sci..

[56] Suzuki T., Fujiwara S., Kinoshita S., Butovich I.A. (2019). Cyclic Change of Fatty Acid Composition in Meibum during the Menstrual Cycle. Invest. Ophthalmol. Vis. Sci..

[57] Suzuki T., Minami Y., Komuro A., Yokoi N., Kinoshita S. (2017). Meibomian Gland Physiology in Pre- and Postmenopausal Women. Invest. Ophthalmol. Vis. Sci..

[58] Fearn S. (2015). An Introduction to Time-of-Flight Secondary Ion Mass Spectrometry (ToF-SIMS) and Its Application to Materials Science.

[59] Anderson M.J. (2001). A new method for non-parametric multivariate analysis of variance. Austral Ecol..

[60] Gretton A., Borgwardt K.M., Rasch M.J., Schölkopf B., Smola A. (2012). A Kernel Two-Sample Test. J. Mach. Learn. Res..

[61] Welch B.L. (1947). The Generalization of Student’s Problem when Several Different Population Variances are Involved. Biometrika.

[62] Cohen J. (1988). Statistical Power Analysis for the Behavioral Sciences.

[63] Pearson K. (1901). On lines and planes of closest fit to systems of points in space. Lond. Edinb. Dublin Philos. Mag. J. Sci..

[64] Steinhaus H. (1957). Sur la division des corps matériels en parties. Bull. L’Académie Pol. Des Sci..

