# Supplementary material for: Composition of Human Meibomian Gland Secretions: Insights from TOF-SIMS Analysis
Source: Int J Mol Sci. 2026 Feb 5;27(3):1590. doi: 10.3390/ijms27031590 (PMC12898181; doi:10.3390/ijms27031590)
Supplement: Supplementary file 1 [file ijms-27-01590-s001.zip › ijms-4069913-supplementary.pdf]

Supplementary information

Composition of Human Meibomian Gland Secretions: Insights from TOF-SIMS Analysis

Katarzyna Balin [1], Beata Węglarz [2,3] Karol Dobiczek[4], and Dorota Tarnawska [2,3,*]

1 A. Chełkowski Institute of Physics, University of Silesia, 75 Pułku Piechoty 1A, Chorzów, 41-500, Poland; katarzyna.balin@us.edu.pl
2 Clinical Department of Ophthalmology, District Railway Hospital, Panewnicka 65, Katowice, 40-760, Poland; weglarz.beata@gmail.com
3 Institute of Biomedical Engineering, Faculty of Science and Technology, University of Silesia in Katowice, 75 Pułku Piechoty 1A, Chorzów, 41-500, Poland; dorota.tarnawska@us.edu.pl
4 Department of Human-Centered Artificial Intelligence, Institute of Applied Computer Science, Jagiellonian University, Łojasiewicza 11, Krakow, 30-348, Poland; karol.dobiczek@gmail.com
* Correspondence: dorota.tarnawska@us.edu.pl;

Table S1. Normalized to total intensity peak areas of characteristic positive ions for the complete cohort of meibum samples.

Ion	H1	H2	H3	H4	H5	D1	D2	D3	D4	D5
PN^+	1.48×10^{-5}	1.42×10^{-5}	3.20×10^{-5}	1.10×10^{-5}	1.19×10^{-5}	3.11×10^{-5}	8.10×10^{-6}	5.33×10^{-5}	1.92×10^{-5}	2.50×10^{-5}
CHO_2^+	3.93×10^{-5}	4.08×10^{-5}	4.69×10^{-5}	4.14×10^{-5}	4.26×10^{-5}	5.74×10^{-5}	4.50×10^{-5}	7.66×10^{-5}	5.28×10^{-5}	8.21×10^{-5}
CH_3NO^+	5.01×10^{-5}	4.32×10^{-5}	1.18×10^{-4}	3.50×10^{-5}	3.08×10^{-5}	1.12×10^{-4}	3.19×10^{-5}	1.28×10^{-4}	3.14×10^{-5}	6.64×10^{-5}
$C_2H_5O^+$	3.61×10^{-4}	3.39×10^{-4}	3.83×10^{-4}	3.56×10^{-4}	3.50×10^{-4}	4.81×10^{-4}	4.21×10^{-4}	5.86×10^{-4}	4.54×10^{-4}	5.89×10^{-4}
$C_2H_7N^+$	6.28×10^{-5}	5.64×10^{-5}	7.08×10^{-5}	5.35×10^{-5}	4.95×10^{-5}	7.40×10^{-5}	7.63×10^{-5}	1.24×10^{-4}	7.14×10^{-5}	1.30×10^{-4}
$C_{15}H_{27}O^+$	2.34×10^{-5}	2.79×10^{-5}	2.91×10^{-5}	2.08×10^{-5}	2.36×10^{-5}	3.25×10^{-5}	1.99×10^{-5}	2.47×10^{-5}	3.11×10^{-5}	2.97×10^{-5}
$C_{16}H_{28}O^+$	1.46×10^{-4}	9.67×10^{-5}	7.40×10^{-5}	1.51×10^{-4}	1.27×10^{-4}	1.27×10^{-4}	3.31×10^{-4}	5.67×10^{-5}	8.98×10^{-5}	1.18×10^{-4}
$C_{17}H_7N_2^+$	5.80×10^{-5}	5.58×10^{-5}	3.49×10^{-5}	5.44×10^{-5}	5.25×10^{-5}	3.99×10^{-5}	5.05×10^{-5}	5.50×10^{-5}	2.77×10^{-5}	2.97×10^{-5}
$C_{18}H_{23}^+$	8.30×10^{-5}	7.29×10^{-5}	1.06×10^{-4}	7.29×10^{-5}	7.78×10^{-5}	5.20×10^{-5}	6.56×10^{-5}	7.38×10^{-5}	6.23×10^{-5}	3.62×10^{-5}
$C_{16}H_{31}O^+$	2.31×10^{-5}	1.96×10^{-5}	3.63×10^{-5}	1.93×10^{-5}	2.13×10^{-5}	4.65×10^{-5}	2.18×10^{-5}	2.34×10^{-5}	4.23×10^{-5}	6.02×10^{-5}
$C_{16}H_{33}O_2^+$	3.71×10^{-4}	4.77×10^{-4}	5.32×10^{-4}	4.80×10^{-4}	5.20×10^{-4}	1.29×10^{-3}	4.62×10^{-4}	5.69×10^{-4}	9.61×10^{-4}	2.41×10^{-3}
$C_{18}H_{32}O^+$	4.32×10^{-4}	4.10×10^{-4}	3.63×10^{-4}	3.69×10^{-4}	3.96×10^{-4}	4.91×10^{-4}	1.10×10^{-3}	1.85×10^{-4}	4.01×10^{-4}	3.52×10^{-4}
$C_{20}H_{38}^+$	3.87×10^{-4}	2.77×10^{-4}	2.22×10^{-4}	1.65×10^{-4}	2.19×10^{-4}	2.32×10^{-4}	6.63×10^{-4}	1.30×10^{-4}	2.43×10^{-4}	1.27×10^{-4}
$C_{21}H_{38}N^+$	2.31×10^{-5}	5.19×10^{-5}	1.05×10^{-4}	5.51×10^{-5}	4.37×10^{-5}	2.10×10^{-5}	1.62×10^{-4}	4.62×10^{-5}	3.20×10^{-5}	8.16×10^{-5}
$C_{24}H_{44}^+$	2.12×10^{-5}	6.23×10^{-5}	1.38×10^{-4}	7.03×10^{-5}	4.31×10^{-5}	2.69×10^{-5}	1.83×10^{-4}	6.43×10^{-5}	2.94×10^{-5}	7.90×10^{-5}

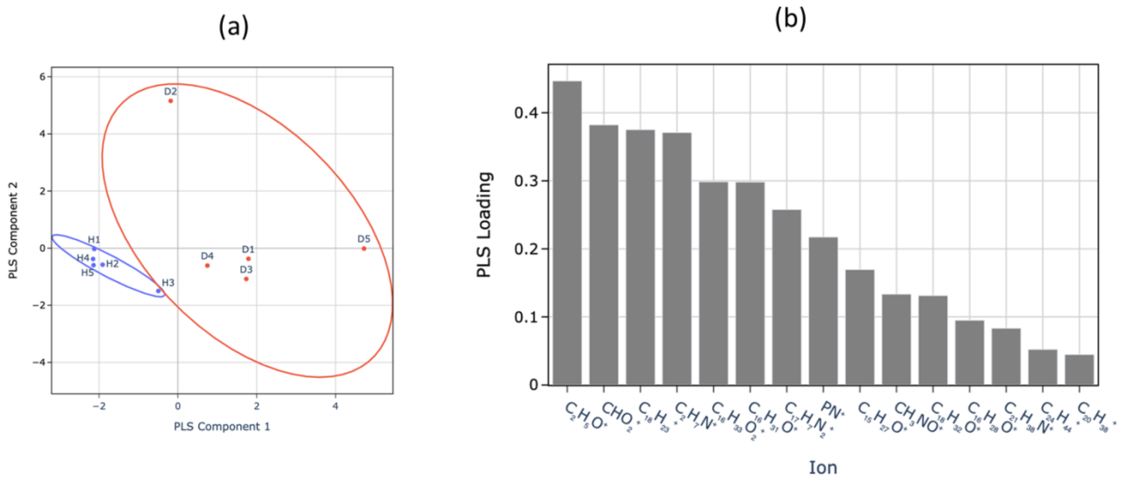

Figure S1 PLS-DA analysis of the data: (a) Data points projected to the two PLS components. The colors represent the two data clusters and the corresponding confidence ellipses. (b) PLS loading scores showing the level of contribution of each ion to the group separation.
